# The flavonoid hesperidin exerts anti-photoaging effect by downregulating matrix metalloproteinase (MMP)-9 expression via mitogen activated protein kinase (MAPK)-dependent signaling pathways

**DOI:** 10.1186/s12906-017-2058-8

**Published:** 2018-01-30

**Authors:** Hee Jeong Lee, A-Rang Im, Su-Man Kim, Hyung-Sik Kang, Jae Dong Lee, Sungwook Chae

**Affiliations:** 1 Department of Microbiology, Disivion of Natural Science, Pusan National University, Busan, 609-735 South Korea; 2 KM Convergence Research Division, Korea Institute of Oriental Medicine, 1672 Yuseongdae-ro, Yuseong-gu, Daejeon, 34054 South Korea; 3 School of Biological Sciences and Technology, Chonnam National University, 77 Yongbong-ro, Buk-gu, Gwangju, 500-757 Republic of Korea; 4 University of Science and Technology, 217 Gajeong-ro, Yuseong-gu, Daejeon, 305-333 South Korea

**Keywords:** Ultraviolet B, Hesperidin, Matrix metalloproteinase-9, Transepidermal water loss, Photoaging

## Abstract

**Background:**

Hesperidin is a flavonoid with antioxidant, anti-inflammatory, and immune modulatory activities. Photoaging is a consequence of chronic exposure to the sun and ultraviolet (UV) radiation. This study was designed to evaluate the efficacy of hesperidin against photoaging of dorsal skin in hairless mice.

**Methods:**

Hairless male mice (6-week-old) were divided into three groups (n = 7): control, UVB-treated vehicle, and UVB-treated hesperidin groups. UVB-irradiated mice from hesperidin group were orally administered 0.1 mL of water containing 100 mg/kg body weight per day hesperidin.

**Results:**

The mean length and depth of wrinkles in the UVB-treated hesperidin group significantly improved after the oral administration of hesperidin, which significantly inhibited the increase in epidermal thickness and epidermal hypertrophy (P < 0.05). UVB irradiation of mice induced epidermal barrier dysfunction including an increase in the transepidermal water loss (TEWL); however, hesperidin decreased the TEWL. UVB irradiation increased the expression of MMP-9 and pro-inflammatory cytokines whereas UVB-treated hesperidin group showed reduced expression. These results indicate that hesperidin showed anti-photoaging activity in the UVB-irradiated hairless mice. In conclusion, hesperidin inhibited the UVB-induced increase in skin thickness, wrinkle formation, and collagen fiber loss in male hairless mice.

**Conclusions:**

These results suggest that hesperidin shows potent anti-photoaging activity by regulating MMP-9 expression through the suppression of MAPK-dependent signaling pathways.

## Background

Aging is a process of progressive reduction in the maximal function and reserve capacity of all body organs including the skin [1]. Skin aging can be classified into two types, intrinsic aging (chronological) and extrinsic aging (photoaging) [2]. Intrinsic aging is a biological process common to all living organisms, and is characterized by an age-dependent deterioration of the skin function and structure, such as epidermal atrophy and dermal-epidermal junctional flattening [3]. In contrast, extrinsic aging (photoaging) results from chronic exposure of the skin to sunlight, and is characterized by histological changes, including damage to collagen fibers and excessive deposition of abnormal elastic fibers [4] UV irradiation induces changes in the physiologic and biochemical features of the skin that lead to increase in the epidermal thickness, skin damage and skin dehydration, and transepidermal water loss (TEWL) [5].

UV irradiation-activated receptors lead to intracellular signaling through stimulation of the stress-associated mitogen-activated protein kinases (MAPK) that regulate the expression of matrix metalloproteinase (MMP)-9 and induce transcriptional factors [6]. UV-irradiated photodamaged skin shows reduced collagen synthesis, and increased levels and activity of MMPs, specifically MMP-1, MMP-3, and MMP-9 [7]. Degradation of extracellular matrix components by MMPs is an important event in common biological processes [8]. For example, MMPs are involved in the extracellular matrix remodeling and play important roles in morphogenesis, angiogenesis, skin ulceration, tumor invasion, and photoaging [9]. MMPs are suggested to be UV-induced aging factors [10]. Even extremely low levels of UVB irradiation can upregulate the MMP activity in human skin. In addition, UV irradiation activates the nuclear factor-kappa B (NF-κB) transcription factor that induces the expression of pro-inflammatory cytokines, proteins involved in immunoregulation, and stimulates the expression of MMPs [11]. UVB-induced inflammatory response is characterized by acute development of edema and erythema, increase in dermal inflammatory cell infiltrates, and augmented prostaglandin synthesis [12]. Moreover, there is an increase in pro-inflammatory cytokines, such as tumor necrosis factor alpha (TNF-α), interleukin (IL)-1β, and IL-8, that accelerates the skin damage and results in MMP activation [13]. In this study, we used hairless mice model for studying photoaging that suitable for studying histological changes of photoaged skin such as wrinkle formation as induced by UVB irradiation [14, 15]. Therefore, the present study examined the effects of the oral administration of hesperidin on UVB-induced hairless mice model including wrinkle formation, MMP-9 expression, and anti-inflammatory effects.

**Fig. 1:**
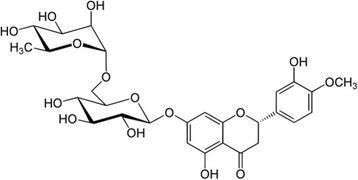
The chemical structure of hesperidin ((2*S*)-5-hydroxy-2-(3-hydroxy-4-methoxyphenyl)-7-[(2*S*,3*R*,4*S*,5*S*,6*R*)-3,4,5-trihydroxy-6-[[(2*R*,3*R*,4*R*,5*R*,6*S*)-3,4,5-trihydroxy-6-methyloxan-2-yl]oxymethyl]oxan-2-yl]oxy-2,3-dihydrochromen-4-one)

Hesperidin [3′,5,7-trihydroxy-4′-methoxyflavanone-7-(6-α-l-rhamnopyranosyl-β-d-glucopyranoside)], a flavanone glycoside comprising an aglycone hesperetin and an attached disaccharide rutinose, is found abundantly in citrus fruits and has been reported to exert a wide range of pharmacological effects [16, 17] (Fig. 1). For example, hesperidin exhibited pronounced anticancer activity against some selected human carcinoma cell lines and showed anti-inflammatory effects [18]. However, little is known about the effect of hesperidin on UVB-induced skin cell or animal model. So, in previous study, hesperidin shielded human keratinocytes from UVB radiation-induced damage and apoptosis via its antioxidant and UVB absorption properties [19]. Also, in many previous studies, the orally administrated compounds are absorbed into the body, so they have a photoprotective effects on UVB-induced animal models [20, 21]. In this study, we used male HR-1 hairless mice to assess the protective effect of hesperidin on photoaging. We examined the effect of hesperidin on UVB-induced photoaging in the skin of hairless mice by evaluating various parameters of photoaging.

## Methods

### Reagents

Hesperidin was obtained from Wako (Wako Pure Chemicals, Osaka, Japan). UVB irradiation was carried out using a UVM-225D Mineralight UV Display Lamp (UVP, Phoenix, AZ, USA). Replicas of mouse dorsal skin were obtained using a Repliflo Cartridge Kit (CuDerm Corp., Dallas, TX, USA). Antibodies against ERK, phospho ERK, MEK, phospho MEK, and anti MMP-9 were purchased from Cell Signaling Technology (Beverly, MA, U.S.A.).

### Experimental animals and oral administration

Male hairless mice (Hos/HR-1, 6-week-old) were purchased from Japan SLC, Inc. (Sizuoka, Japan). Mice were housed in a climate-controlled room at 24 °C and 50% humidity under a 12 h light/dark cycle. They were acclimatized for 1 week prior to the study, and provided free access to food and water. They were divided into 3 groups randomly (*n* = 7 per group): control, UVB-treated vehicle, and UVB-treated hesperidin (100 mg/kg) groups. Hesperidin treated group was orally administered 0.1 mL of water containing 100 mg/kg body weight per day hesperidin in the study period (5 days a week for 12 weeks). Animals in the vehicle group were orally administered drinking water, whereas the unexposed control group animals did not receive any treatment. All experimental protocols were approved by the Korea Institute of Oriental Medicine Institutional Animal Care and Use Committee (12–045).

### UVB irradiation in mice

This experiment investigated the effect of orally administered hesperidin on the dorsal skin of UV-irradiated mice. Mice were irradiated 3 times at 48 h intervals per week for 12 weeks. The amount of irradiation was progressively increased from 60 mJ/cm^2^ per exposure at week 1 to 90 mJ/cm^2^ at week 7.

### Evaluation of wrinkle formation and TEWL

Replicas of mouse dorsal skin were obtained using Repliflo Cartridge Kit (CuDerm Corp., U.S.A.). A dorsal skin sample was taken after the animals were sacrificed. Animals were sacrificed by lethal inhalation of carbon dioxide. The impression replicas were set on a horizontal sample stand, and wrinkle shadows were produced by illumination with a fixed-intensity light at 35° angle using an optical light source. Black and white images were acquired with a CCD camera and analyzed by Skin-Visiometer VL 650 software (Courage & Khazhka, Cologne, Germany). The parameters used in the assessment of skin wrinkles were the average length and average depth of wrinkles. For the evaluation of skin hydration, TEWL, as a marker of epidermal skin barrier function, was measured with Tewameter® TM300 mounted on a Multi Probe Adapter (CK Electronics GmbH, Germany).

### Histological Examination

Doral skin was fixed in 10% neutral buffered formalin, embedded in paraffin, and sectioned at 5-μm thickness. Skin sections were stained with hematoxylin and eosin (H&E). The thickness of the epidermis was measured under light microscopy with an eyepiece micrometer (Olympus, Japan).

### Immunohistochemistry

After taken of dorsal skin, all dorsal skin was snap frozen in nitrogen and stored at −80 °C for analysis. The skin tissues were fixed in 4% paraformaldehyde, embedded in paraffin, and sectioned at 10-μm thickness using a microtome. After deparaffinization and rehydration, the sections were incubated with 5% BSA (in PBS) at room temperature for 1 h to block non-specific binding. Sections were then incubated with following primary antibodies: anti-TNF-α (1:200; Abcam, Cambridge, MA) and anti-IL-8 (1:200; Immuno-biological laboratoriesCo, Fuhioka, Japan) in a humidified chamber overnight at 4 °C. After washing, the tissue sections were incubated with appropriate fluorescence conjugated Alexa 488 (1:2000; ThermoFisher Scientific, Waltham, CA) secondary antibodies for 1 h and counter-stained with DAPI (ThermoFisher scientific) for 15 min. Subsequently, the sections were washed, mounted using Vecta shield mounting media (Vector Laboratories, Burlingame, CA), and imaged using a fluorescence microscope (Leica, Wetzlar, Germany).

### Preparation of skin lysates

At the end of the experiments, mice were sacrificed by cervical dislocation, and the dorsal skin was excised. The fat was removed, and the skin was immediately pulverized with liquid nitrogen using a mortar and pestle. The pulverized skin was homogenized on ice with Precellys®24 (Bertin, USA) tissue homogenizer. Subsequently, proteins were extracted with 20% SDS solution containing 1 mM phenylmethylsulfonyl fluoride (PMSF), 10 mM iodoacetamide, 1 mM leupetin, 0.1 mM sodium orthovanadate, and 5 mM sodium fluoride. The obtained lysates were centrifuged at 13,000 rpm for 30 min, and the protein content in the supernatant was determined using a Bio-Rad protein assay kit (Bio-Rad).

### Gelatin zymography

To assess the gelatinolytic activities of MMP-9, equal amounts of the protein extract were mixed with non-reducing sample buffer, incubated for 10 min at room temperature, and subjected to gelatin zymography (0.1% gelatin) under non-reducing conditions. After electrophoresis, the gel was washed with 2.5% Triton X-100 for 1 h to remove SDS, and then incubated for 24 h at 37 °C in developing buffer (1 M Tris-HCl, pH 7.5, 10 mM CaCl_2_). Subsequently, the gels were stained with Coomassie brilliant blue followed by destaining (25% ethanol, 8% acetic acid). Gelatinolytic activity could be observed as horizontal white bands on a blue background. Relative band densities were analyzed using Image J 1.44 software (NIH, Bethesda, MD, U.S.A.)

### RNA extraction and quantitative real-time polymerase chain reaction

Total RNA was extracted from UVB-irradiated mouse skin tissue using the TRIzol reagent and the protocol recommended by the manufacturer (Invitrogen, Carlsbad, CA, USA). Quantitative real-time polymerase chain reaction (qRT-PCR) was performed using TaqMan assays (Applied Biosystems, Foster City, CA, USA) specific for MMP-9 on a QuantStudio™ 6 Flex Real-Time PCR system (Applied Biosystems). Each sample was assayed in triplicate, and relative mRNA expression levels were calculated using the ΔΔCt method and normalized to the β-actin mRNA level in each sample.

### Western blotting

The lysates prepared from 100 μg of the fat-removed dorsal skin of mice, were centrifuged at 13,000 rpm for 30 min, and aliquots of the supernatant containing 100 μg of proteins were subjected to 10% SDS-PAGE. After electrophoresis, the proteins were blotted onto nitrocellulose membrane. Subsequently, the membranes were blocked by incubation with TBS-T (0.1% Tween 20) containing 5% BSA. The membranes were then incubated with primary antibodies (anti-phospho-ERK, anti-phospho–MEK, anti-MMP-9, or anti-ERK), and washed with TBS-T. Protein bands were visualized using a chemiluminescence detection kit (Amersham Pharmacia Biotech) after hybridization with horseradish peroxidase (HRP)-conjugated secondary antibodies (goat anti-rabbit IgG or goat anti-mouse IgG Ab diluted 1:1000). The relative amounts of proteins were detected using an enhanced chemiluminescence Western Blotting Detection Kit (Amersham, Little Chalfont, Buckinghamshire, UK).

### Statistical analysis

All measurements are presented as means ± S.D. values of triplicate. Differences were a *p* value of <0.05 was considered significant.

## Results

### Hesperidin inhibits UVB-induced wrinkle **formation in mouse skin**

**Fig. 2:**
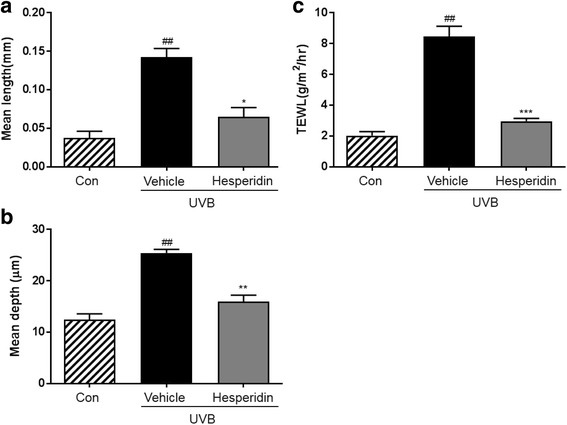
Effects of hesperidin on the UVB-induced wrinkle formation in hairless mice and TEWL. Effects of hesperidin on the (**a**) mean length and (**b**) mean depth of skin wrinkles, and (**c**) TEWL. ##*P* < 0.01 significant difference from control and *P < 0.05, **P < 0.01, and ***P < 0.001 significant difference from UVB/vehicle groups

To investigate the effect of hesperidin on UVB-induced wrinkle formation in vivo, a photoaging study was performed using an HR-1 hairless mouse system. Repeated exposure of the mouse dorsal skin to UVB radiations over 12 weeks resulted in wrinkle formation, which was prevented by hesperidin (Fig. 2). These results indicate that hesperidin significantly attenuates the UVB-induced photoaging and wrinkle formation.

### Wrinkle measurement and analysis of replicas

The skin replicas were analyzed using an image analysis system to quantify the degree of wrinkle formation. The mean length and depth of wrinkles in the UVB-treated vehicle group were significantly higher than those in the unexposed control group (Fig. 2a and b). Hesperidin treatment significantly improved the mean wrinkle length and depth in the UVB-treated vehicle group. Furthermore, it improved the UVB-induced hydration changes, skin water-holding capacity, and TEWL in the UVB-irradiated hairless mice (Fig. 2c). TEWL tended be higher in the UVB-treated vehicle group than in the control group. Importantly, it was lower in the UVB-treated hesperidin group than in the UVB-treated vehicle group.

### Effect of hesperidin on the thickness of the epidermis and histological observations in the UVB-irradiated hairless mice

**Fig. 3:**
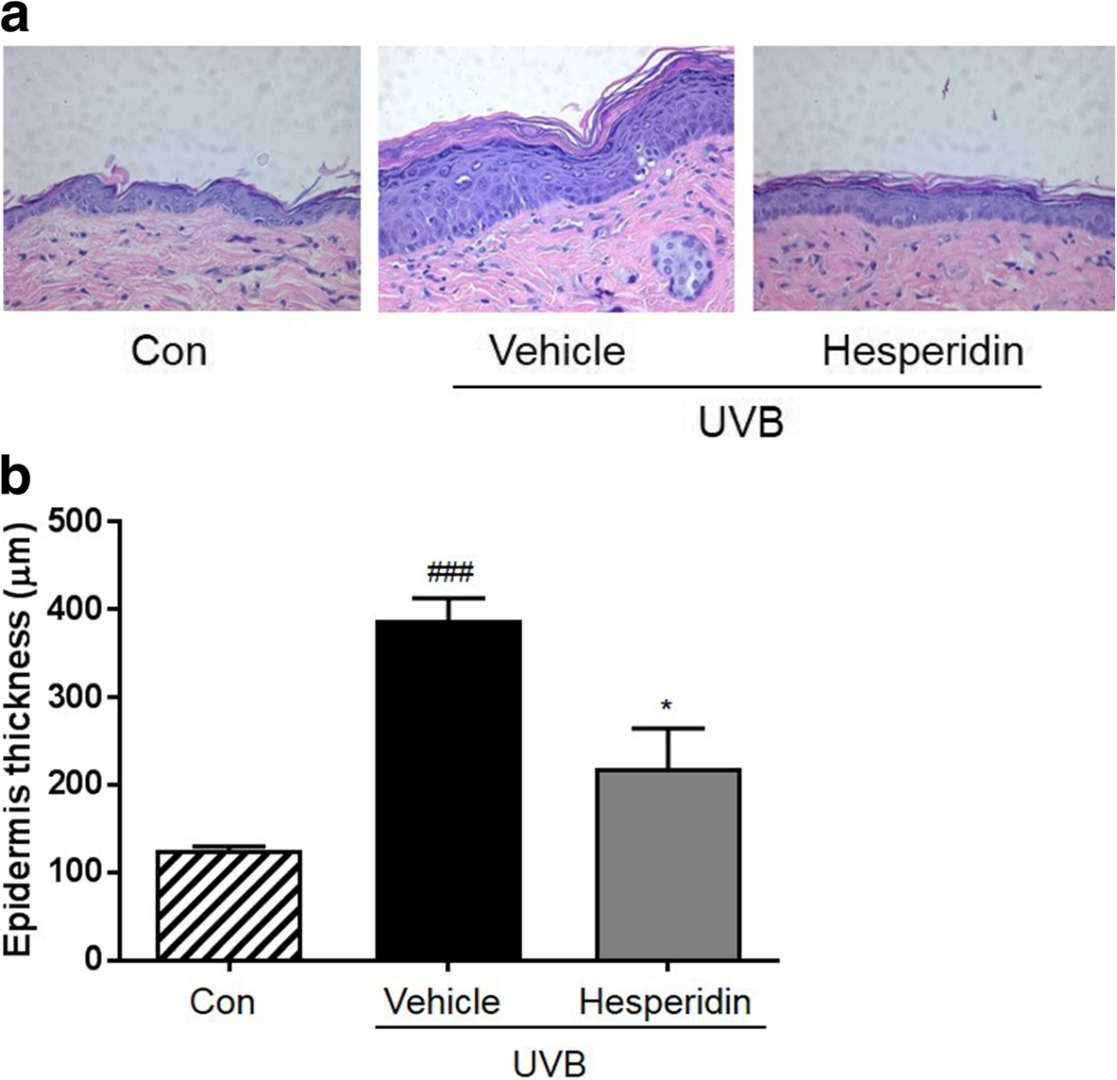
Effect of hesperidin on the UVB irradiation-induced epidermal thickening in hairless mice dorsal skin. (**a**) Hematoxylin- and eosin-stained images of UVB-irradiated mice skin. Images are representative of results from 5 tissue samples. (**b**) Hesperidin improved the UVB-induced increase in epidermal thickness. ###*P* < 0.001 significant difference from control and *P < 0.05 significant difference from UVB/vehicle groups

The thickness of the epidermis of the dorsal skin was significantly increased by UVB irradiation as shown by the H&E staining. Hesperidin treatment significantly inhibited the increase in epidermal thickness (Fig. 3a). Furthermore, hesperidin treatment significantly reduced epidermal hypertrophy (Fig. 3b).

### Hesperidin inhibits UVB-induced MMP-9 expression and activity

**Fig. 4:**
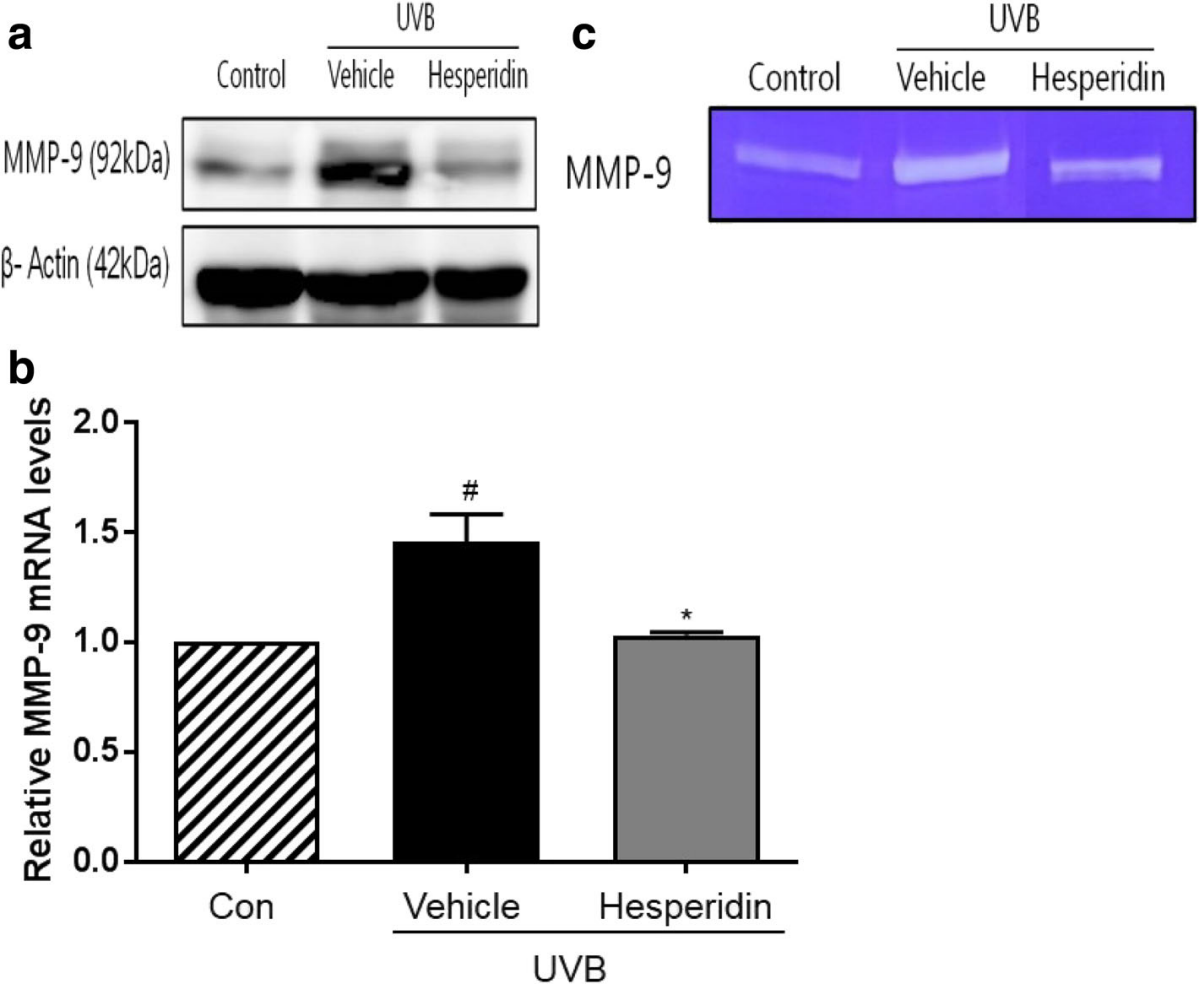
Inhibitory activity of hesperidin on the UVB-induced MMP-9 protein expression and enzyme activity in the photoaged hairless mice model. (**a**) Inhibition of the UVB-induced MMP-9 protein expression by hesperidin. The MMP-9 levels were determined by western blotting analysis. (**b**) The mRNA MMP-9 levels were determined by RT-PCR. (**c**) Inhibition of MMP-9 activity was measured by gelatin zymography. #*P* < 0.05 significant difference from control and *P < 0.05 significant difference from UVB/vehicle groups

The inhibitory effect of hesperidin on the UVB-induced MMP-9 expression was evaluated by western blot analysis and gelatin zymography, and RT-PCR. Western blotting analysis revealed that hesperidin inhibited the UVB-induced expression of MMP-9 protein (Fig. 4a). Also, in Fig. 4b, MMP-9 mRNA levels were increased in cells exposed to UVB and suppressed by treatment with hesperidin. Moreover, hesperidin suppressed the MMP-9 activity as shown by gelatin zymography (Fig. 4c).

### Hesperidin inhibits UVB-induced pro-inflammatory cytokines

**Fig. 5:**
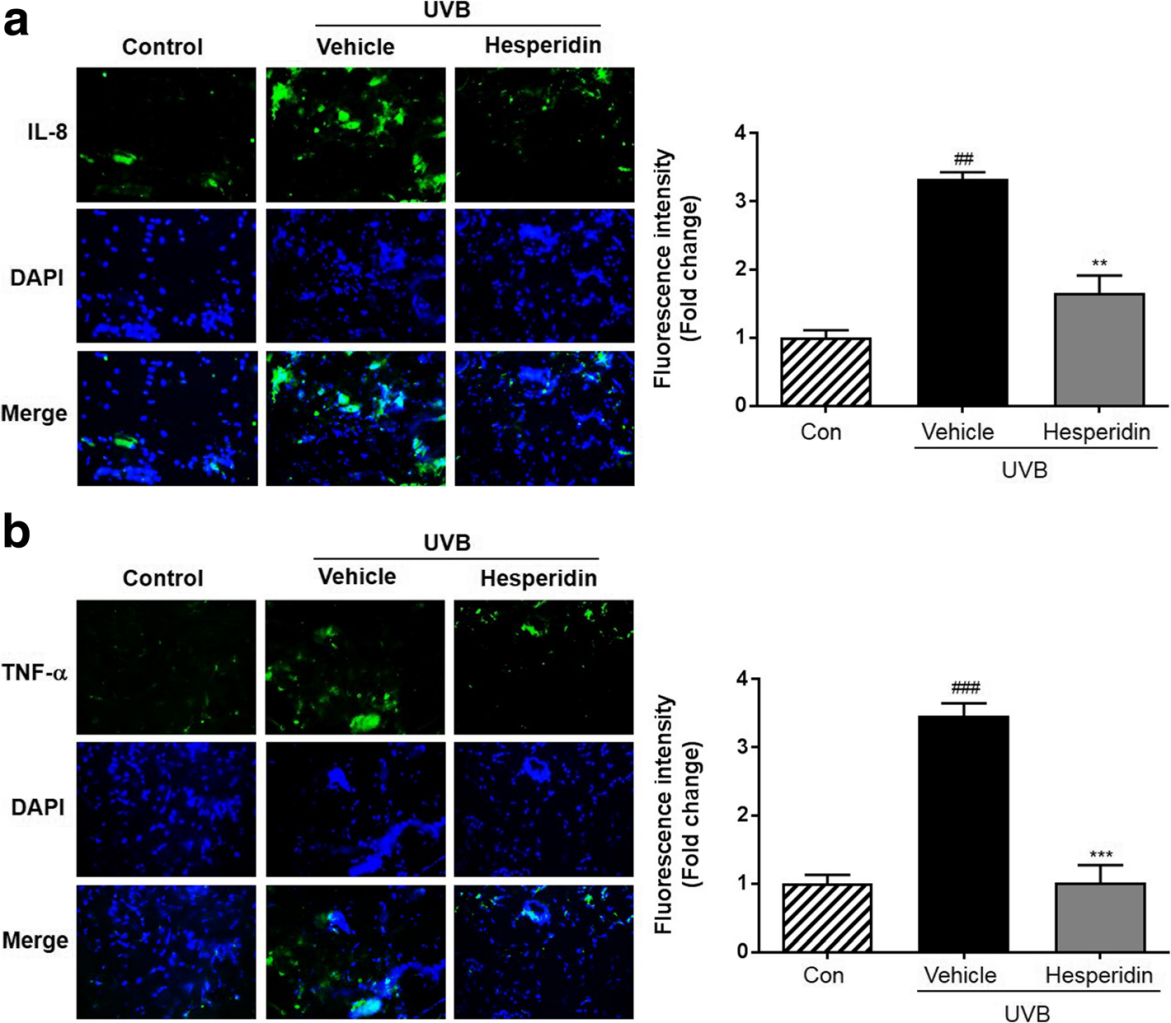
Images of immunohistochemically-stained skin tissue. Immunofluorescence study was carried out using (**a**) IL-8 and (**b**) TNF-α antibodies. Blue staining represents nucleus. Quantitative analysis of fluorescence intensity was performed by Image J software. ##*P* < 0.01, and ###*P* < 0.001 significant difference from control and **P < 0.01, and ***P < 0.001 Significant difference from UVB/vehicle groups

The results of in vivo study on skin tissue showed that the production of pro-inflammatory cytokines including IL-8 and TNF-α was enhanced in the UVB-exposed skin tissue (Fig. 5). Moreover, a significant increase in the production of TNF-α and IL-8, measured in relative fluorescence units, was observed in the UVB-irradiated mice compared to that in the control mice. Next, the distribution of pro-inflammatory cytokines in the skin was analyzed. It was observed that compared to the control mice, the UVB-irradiated mice had significantly increased production of inflammatory cytokines in the skin. This suggested that hesperidin exerted a photoprotective effect by reducing the inflammatory response to UVB radiation.

### Inhibition of the UVB-induced phosphorylation of MEK and ERK by hesperidin

**Fig. 6:**
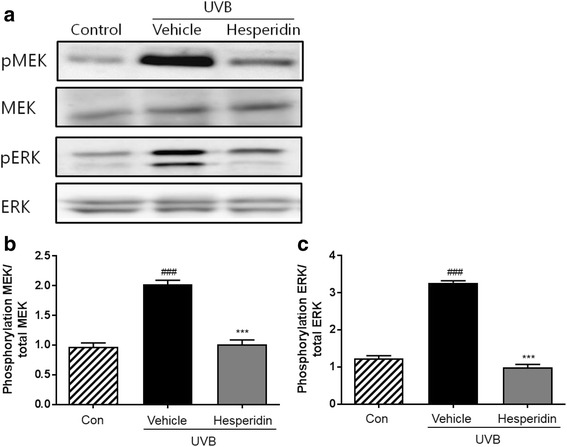
Effects of hesperidin on the phosphorylation of MEK and ERK in the UVB-irradiated mice model. (**a**) Hesperidin inhibited the phosphorylation of MEK and ERK. Western blot analyses of phospho-MEK, MEK, phospho-ERK, and ERK expression were performed. Quantitative analysis of intensity was performed by Image J software on (**b**) phospho-MEK and (**c**) phospho-ERK. ###*P* < 0.001 significant difference from control and ***P < 0.001 significant difference from UVB/vehicle groups

Western blot analysis showed that UVB exposure led to the phosphorylation of MEK and ERK (Fig. 6). Therefore, the effect of hesperidin on UVB-induced phosphorylation of MEK and ERK was evaluated. Results showed that hesperidin treatment decreased the expression of MEK and ERK in UVB-irradiated mice. These data confirm that hesperidin protects the mice dorsal skin from UVB-induced damage.

## Discussion

It is well known that UV radiations from sunlight are a major environmental factor that cause acute and chronic changes in the human skin [22]. Chronic exposure to UV radiation is known to be a primary cause of skin photoaging, which is characterized by skin wrinkles, roughness, laxity, irregular pigmentation, telangiectasia, atrophy, and neoplasia [23].

Hesperidin is a flavanone glycoside abundantly found in sweet orange and lemon [17]. It has a wide range of biological effects, including attenuation of UVB-induced apoptosis mediated by mitigation of oxidative stress in human keratinocytes [24]. Furthermore, it has been reported that phytochemicals such as proanthocyanidin, genistein, and daidzein are potential photoprotective agents against UVB-induced skin damage [25, 26]. Previous studies have shown that flavonoids can be used orally to protect the skin from UVB-induced damage [27, 28]. For example, quercetin inhibited the UV irradiation-induced inflammatory cytokine production in primary human keratinocytes by suppressing the NF-κB pathway [29]. Furthermore in this study, we demonstrated that the natural flavonoid hesperidin exerts anti-photoaging effect by activating MMP-9 expression via the MAPK signaling pathways on UVB-induced hairless mice model. In this study, we investigated the anti-photoaging effects of hesperidin against UVB-irradiation on the dorsal skin of HR-1 mice. Histological studies have shown that photoaging of the skin is associated with increased epidermal thickness and alterations in the connective tissue organization [30]. Similarly, we found that chronic UVB irradiation of the mice dorsal skin induced wrinkle formation and epidermal thickening. Hesperidin improved these damages induced by UVB.

Epidermal thickness is used as a quantitative parameter for assessing inflammation and skin photoaging [31]. UVB irradiation causes skin damage, leading to skin dehydration and an increase in TEWL [32]. Increased TEWL impairs enzymatic functions that result in the visible appearance of dry and aged skin [33]. Because UV irradiation of the skin disrupts the epidermal permeability barrier function, TEWL is an important parameter to be assessed in the studies of skin photoprotective effects [34]. Hesperidin treatment effectively reduced skin damage in this study. This was associated with an increase in skin hydration and decreases in TEWL.

MMPs are zinc-dependent endopeptidases associated with extracellular matrix remodeling that play important roles in morphogenesis, angiogenesis, arthritis, skin ulceration, tumor invasion, and photoaging [35]. MMP expression induced by UVB exposure leads to the degradation of the extracellular matrix, including collagen fibers, and thus contributes to skin wrinkle formation [36]. UVB irradiation is specifically associated to the expression of MMP-1, −3, and −9 in the normal human epidermis in vivo, which indicates that MMPs are the main UVB-induced aging factors [10, 37]. UV irradiation of the skin leads to MMP-9 formation, which degrades collagen type IV, an important component of the basement membrane in the skin dermal epidermal junction [38, 39]. Hesperidin ameliorated the photoaging in the dorsal skin of mice by downregulating the expression and activity of MMP-9.

UVB-induced cytokines act in a cascade to induce inflammation. They are initially released by keratinocytes or inflammatory cells in the skin after UVB irradiation, and subsequently synergize with UV-irradiated keratinocytes to further increase their production [40]. TNF-α, and IL-8 are important regulators of the intensity of inflammatory reactions that occur during the host response to injurious stimuli such as UVB radiations that cause severe sunburn reaction [41]. MAPKs are known to regulate the expression of MMP-9, it showed that the inhibition of MMP-9 expression in human dermal fibroblasts was mediated by inhibition of the MEK/ERK signaling pathway [6]. It has been reported that the activation of MEK/ERK signaling is responsible for the induction of MMP-9 and is required for responses in epidermal keratinocytes [42]. Therefore, the UVB-induced phosphorylation of MEK/ERK may be an important molecular target for controlling the MMP expression by anti-photoaging treatments. Results of this study showed that hesperidin attenuated the UVB-induced MMP-9 expression regulated by the MEK/ERK pathway.

## Conclusion

In summary, our results showed that hesperidin inhibited the MMP-9 related signaling pathway activated by UVB irradiation. Furthermore, hesperidin prevented the UVB-induced skin thickening, wrinkle formation, and inflammation. Elucidation of the detailed mechanism of MMP-9 inhibition requires further investigation. It can be suggested that hesperidin could be a good candidate as a photoprotective agent for skin care.

## Abbreviations

MAPK: Mitogen-activated protein kinase
MMP: Matrix metalloproteinase
TEWL: Transepidermal water loss
UV: Ultraviolet
